# On Structural Changes in Human Enamel—With Special Reference to Clinical Observations on Hard and Soft Enamel

**Published:** 1898-12

**Authors:** J. Leon Williams

**Affiliations:** London, England.


AMERICAN JOURNAL

—OF—

DENTAL SCIENCE.

VOL. XXXII. BALTIMORE, DECEMBER, 1898. No. 8.

### Selections.

ARTICLE I.

### ON STRUCTURAL CHANGES IN HUMAN ENAMEL.—WITH SPECIAL REFERENCE TO CLINICAL OBSERVATIONS ON HARD AND SOFT ENAMEL.

J. LEON WILLIAMS, D. D. S.. L. D. S., F. R. M. S., LONDON, ENGLAND.

Abstract of a paper read before the British Dental Association, at Bath, England, May 30, 1998.

Mr. President and gentlemen of the British Dental Association, I thank you for your kind reception, and for the honor conferred in the invitation to read a paper before this society.

The wise practical rule you have adopted of limiting papers to twenty minutes makes it unnecessary for me to apologize for not indulging in any purely ornamental introductory remarks, and I shall, I am sure, be acting in accordance with your wishes if I plunge at once into my subject.

An examination of the literature of dentistry will show that the opinion has been held, almost throughout the history of the profession that human teeth undergo structural changes. For a time this belief was confined to the view that teeth degenerate and become softer, and this phase of belief was founded upon clinical observations. But out of these observations there was evolved a theory that the structural changes were due to intra-vitem states, involving fluctuations in the nutrition of the tissues and the general physiological conditions of the parts. Having once got hold of this theory, it was, of course, a perfertly natural thing to go a step farther and say that inasmuch as it is a matter of clinical observation that enamel may become softened, which softening (here the theory stepped in) is due to lack of proper nutrition, therefore the converse must be true, and enamel which has softened or which was not originally properly hardened because of insufficient nutritive material may become hard and resistant if the proper food be supplied. Thus was ushered in the era during which children, and often adults, were dosed with various phosphatic preparations, until many an individual had swallowed material enough to make several sets of teeth, the only noticeable effect of which was, in a great majority of cases, to disturb the digestive functions, and so hasten the destruction of the fast disappearing dental organs. But in some cases, no doubt, the general health was improved by this treatment, and decay of the teeth proceeded more slowly, not because the enamel of the teeth had improved in quality, but because those environing conditions which constitute the true disposing cause of dental caries had been changed by the improvement in the general health. You know how it used to be in a country neighborhood when some one held a successful lottery ticket. It became the talk of the town, and every body invested in lottery tickets. The ninety and nine who lost their money said little or nothing about it, but they all continued to talk of the successful one. And so it was for many years with the administration of phosphatic remedies for decaying teeth. Everybody talked about the few cases which seemed to improve under the treatment, and forgot all about the ninety and nine who were made worse.

More than fifteen years ago I pointed out that, from the priori standpoint, physiological changes in completely formed enamel are impossible. I called attention to the well-known fact that the nutrient function is simply the formative function reduced in activity to the point of making good the loss occasioned by waste, and that, as the formative organ of enamel completely disappeared at or before the time of the uruption of the tooth, there was no possible means by which the enamel molecule or molecules could be formed. The position was a perfectly sound one logically, but, as it made no attempt to explain the softening of teeth, which was a matter of general observation, it failed to carry conviction to the majority. I was severely attacked, as young men must always expect to be when they have the effrontery to present anything new which calls for a modification of older views, and told that I was attempting to teach before I had learned half the alphabet. I have no doubt there was considerable truth in what my opponents said, but still I believed that I had got hold of some valuable facts, and I stuck to them. Ten years or more after I had taken up this position, Dr. G. V. Black, who had throughout supported me., astounded the dental world by the publication of a series of papers in which he showed by a very exhaustive series of experiments that there is no such difference in the per cent. of lime-salts in teeth as had always been supposed. This, at a single stroke, was eliminated what had always been regarded as the chief factor in the problem of tooth degeneration and decay. A little later came the publication by Mr. Charles Tomes of the results of his researches in this same field, which largely confirmed the positions of Dr. Black and myself.

Mr. Tomes was the first to confirm by chemical analysis my contention that there is practically no organic matter in mature enamel, and the absence of organic matter, of course, means an entire absence of organic change. Soon after the publication of Mr. Tomes' report my papers on "Pathology of Enamel" appeared in the "Dental Cosmos." This work was undertaken to clear up the mystery or obscurity surrounding the very commencement of the process of caries on the surface of the tooth. Incidentally several other things appeared, the most important of which was a complete explanation and demonstration of the long observed softening of enamel, even when there is no external evidence that such a softening process is going on. This point was hardly more than touched upon in the series of papers referred to, and, although Dr. Black and a few others seized upon its significance at once, I believe its importance has been generally overlooked, and it is to further and more detailed proof of the phenomena of the softening of enamel by external agencies that I now invite your attention.

If we grind a thin section of normal human enamel and mount it for microscopical examination, under low and medium powers of magnification the enamel-rods appear to be quite smooth, and the individual rods not strongly differentiated from each other. Under high powers the rods are seen to have an irregularly wavy or varicose outline. If this same section, which we will suppose has been temporarily mounted in glycerol, be now exposed for a few minutes to the action of a one-half of one per cent. solution cf hydrochloric acid and again mounted for study, some very marked changes will be observed to have taken place. Not only are the rods much more clearly differentiated from each other, but many of the rods are further seen to be divided into sections. What has happened to produce this appearance? I have said in former papers that the appearance is caused by the partial solution of a cement-substance, which unites the rods and the sections of which the rods are built up. Von Ebner stated some time ago, as you remember, that acids first act upon the substance of the rods themselves, and recently, from several quarters, this matter has been said to be in doubt. Dr. Biro, for instance, in a paper read a short time ago before the Royal Hungarian Society of Doctors, expressed himself as unable to decide whether Von Ebner's statement or mine is to be received. From my point of view, it should not be considered a controversial question for a moment. Any one who has a microscope, and cares to spend twenty minutes' time over the matter, can easily determine the point for himself. Grind a longitudinal section of enamel so that one edge is very thin and the other a trifle thicker, and expose to the action of weak hydrochloric acid for a few minutes and then mount for examination. If the work has been properly carried out, the appearance will always be as stated. The acid, acting on the thin edge of enamel, has completely isolated the ends of many of the rods by dissolving away the cement-substance which united them, and at the same time the globular sections of which the rods are built up are plainly indicated. I do not see how a more complete or convincing proof could be asked for than that which is shown here.

Where the ends of the enamel-rods are shown under much higher power, it is seen that substantially the same effect is produced by the action of the acid excreted by bacteria. The center of the rod is never hollowed out or depressed, but, on the contrary, always projects considerably beyond the cement-substance, thus proving conclusively that the cement-substance is more quickly acted upon than the substance of the rod itself. It is important that the point be thoroughly established, for without it none of the appearances which we may observe in decaying enamel are in the least intelligible; while if it is once clearly seen how acids act upon enamel, all that follows is perfectly plain. We see, then, that the effect of acid artificially applied to enamel is by solution of the cement-substance to render the tissue porous and friable, and consequently less resistant to pressure or to the action of cutting-instruments.

We have now to inquire to what extent a similar process is going on in the enamel when in its natural condition in the mouth. Many of you will remember that in my recent series of papers on "Pathology of Enamel" I published quite a number of photographs showing acid-forming bacteria attached to the surface of enamel. Further experience has convinced me that the cases in which the approximal surfaces are not covered by a thick, felt-like, and glutinous mass of mibro-organisms are very rare. In fact, since I devised a simple means of grinding thin sections of enamel without disturbing any bacteria which might be present on the surface, I have not once failed to find them on the approximal surfaces of teeth. In cases where the enamel is much discolored and it is desired to show the structure of these discolored portions, the section must be cleared and mounted in balsam. The shrinkage which this treatment seems to produce in organic matter almost invariably causes the film of bacteria to become loosened from its attachment to the enamel, and it is thus washed away. As the custom has always been to mount sections of teeth in balsam, this probably explains why the almost universal presence of bacteria on approximal surfaces has not been discovered before.

My studies of this subject during the past year have been chiefly directed toward observing the changes which occur in enamel beneath these bacterial growths, previous to the commencement of the actual breaking down of the tissue. The result of these studies has been surprising, notwithstanding my previous experience. In the clinical examination of the approximal surfaces of teeth, if there is no perceptible roughness, if our fine exploring points pass smoothly over the entire surface, we pronounce the tooth quite sound at that part. I shall be able to show you that this soundness is very often only apparent, and that the actual breaking down of the enamel is only one step coming toward the close of a long series of structural changes, which may have been in progress for years before there is any external evidence of such changes.

The appearances produced by the first action of the acid of bacteria upon enamel vary considerably. This variation is, without doubt, to some extent due to variations in the structure of the tissue, but, I believe, it is largely due to variations in the energy of the micro-organisms themselves. My reason for this view will appear as we proceed.

As there is nothing in the structure of the tissue in these different specimens to account for the vary marked difference in appearance, especially the presence of the light and dark bands, have we any explanation to offer? I think so; and I believe this explanation is one of the most important points I have to present for your consideration, as bearing directly upon the true predisposing cause of dental caries. It has been a matter of well-known observation for many years that rapid decay is accompanied by little or no discoloration,while the tooth-substance in decay is deeply pigmented. I think we may safely assume, that the discolored bands referred to represent a partial arrest in the progress of the action of the acid of decay, or, in other words, the bacteria which produce the acid were, for some reason, less active during a period corresponding to the formation of the dark bands. It would be difficult to prove this view completely without a long series of experiments and observations, but there is some evidence bearing directly upon the point. In my papers on "Pathology of Enamel" I called attention to several specimens in which there was clearly seen a partial or temporary arrest of decay at well-marked lines of stratification. Since the publication of those papers the facts stated on this point have been corroborated by further observations, and I have noticed that in every instance the line along which there is a temporary arrest of decay is deeply colored. But in these instances there is no reason for assuming any decrease in the activity of the bacteria; and this fact led me to look more carefully into the cause of the staining of enamel during the progress of caries, and I find that the discoloration, at least so far as enamel is concerned, is largely an optical illusion. It seems that the structural changes in slow decay are markedly different from those which take place in rapid decay. In rapid decay the acid penetrates quickly and deeply along the lines of the inter-rod cement-substance, and the rods themselves become isolated and break down before there are any very marked changes in their internal structure. But in slow decay the entire substance of the rod becomes infiltrated with acid, and a sort of spongy-like structure is produced, the effect of which is to break up and disperse certain of the constituents of the rays of light so that, viewed by transmitted light, there is an appearance of discoloration; while these same specimens, seen by reflected light, are chalky white.

Now, I find that in all cases the structure in the discolored bands approximates closely to that shown in the case of known slow decay, while the light bands always correspond in structure to that shown in the case of known rapid decay. We thus have a double line of evidence, you see, that the dark bands represent a retardation in the progress of decay. This retardation in those very exceptional cases of marked stratification is probably largely due to the break in the continuity of the cement-substance, but, in all cases where there is no discoverable irregularity in the natural structure of the enamel we may, as I have said, safely assume that the retardation in the action of the acid is due to a change in the environment of the teeth, which is unfavorable to the development and activity of the bacteria producing the acid.

I suppose the strangest thing, and therefore the thing most difficult for those to realize who have not especially studied the subject, is the extent to which the enamel may be penetrated by the acid of decay before there is any indication to the unaided eye of failure on the surface of the tooth.

In cutting into such areas there is small reason for wonder that dentists, with no knowledge of what had really taken place, should have concluded that some important vital change had occurred in the enamel, whereby the tissue had been deprived of a portion of its lime-salts. A degeneration and a softening has taken place, lime salts have been removed, but, as you now see, this has been caused entirely by external agencies; and thus my a priori logical position of long ago, that vital changes cannot occur in the enamel of an erupted tooth, is shown to be in complete harmony with the scientifically demonstrated facts.

In connection with the supposed relation between the presence of bacteria at the commencement of decay and congenital defects in enamel, I may at this point call your attention to the fact that bacteria are most frequently found to be acting upon enamel where the tissue is usually most perfect. It so happens that this is true with reference to that portion or zone of a tooth which includes the approximal surfaces. Aside from those coarser imperfections, the the crevices and sulci on the grinding-surfaces, defective enamel is most frequently found upon the cusps of teeth, where they rarely decay. That is, of course, purely a coincidence, but it is a coincidence worthy of mention.

Even with many who are fully cognizant of the role which bacteria play in causing decay of the teeth, it has been believed that congenital defects are second only in importance to the micro-organisms. Bacteria undoubtedly avail themselves of defects where they exist, but at the same time they act quite independently of all defects. The sheltered situation of an approximal surface, with enamel of perfect structure, is evidently a more favorable condition for decay than almost any form of defect upon the lingual or buccal surfaces of the teeth.

I have frequently referred to the action of the acid of decay as forming canals in the cement-substance between the enamel-rods. There are several very interesting questions relating to the presence of these canals which it may be well to consider a little further. I am convinced that the canals which several observers have seen in human enamel, and referred to as congenital defects, are, in the great majority of cases, the work of bacteria. There are several good reasons for this opinion. Such canals are rarely found in the teeth of wild animals, and in human teeth much less frequently upon the buccal and lingual surfaces than on the approximal surfaces, which are nearly always covered by bacteria. Again, in nearly all instances, as I have previously observed, there are other and unmistakable indications of the depth to which acid has penetrated in enamel; and these other appearances, when compared with the depth of the canals, are found nearly always to correspond exactly. Furthermore, if canals do exist as congenital defects we should, according to the latest researches of Mr. Tomes, expect to find such canals in the substance of the enamel-rod. Now, in the specimens which I am describing this never occurs.

For the benefit of those who still believe that there are fine fibers of organic matter in enamel (from a recent article in the Dental Cosmos I see that there are such believers), and that these canals might possibly have contained organic matter, I will repeat what I have previously stated, that there is not the slightest difficulty in demonstrating the presence of organic matter in enamel when it exists. I have many specimens of the tubular enamel of marsupials in which the organic fibers in the tubes are deeply stained, but in no instance have I ever been able to demonstrate the presence of stainable matter, other than bacteria, in human enamel. The evidence, therefore, seems to be nearly all in favor of the view that none of the canals which I have described are due to congenital structural defects or peculiarities, but are produced by the acid excreted by bacteria. That the most of them are so produced is beyond question. In a former paper I published a photograph which showed that the entire surface of a tooth may become covered with bacteria, and the whole enamel covering thus become softened before there is any external cavity formed.

And now we come back to the bearing of all these facts, which I have been exhibiting to you, on our clinical observations. We see clearly that the long-established belief in the softening of enamel, even when there is no external evidence of decay, is well founded. Enamel which at one time offered great resistance to cutting-instruments may be observed a few years later, to use a somewhat exaggerated but frequent expression, to cut almost as easily as chalk; and these teeth not only cut easily, but, by the ordinary reflected light in which they are seen, they have a chalky look to the eye. This appearance invariably follows the application of acid to enamel. The deduction from these clinical observations that some intra-vitem change had taken place in the tissue merely represents the everlasting desire of the human mind for an explanation.

I may add, in this connection, that I regard the claim that the enamel may harden after having once been softened as without the smallest foundation. I say this not alone because an explanation of such a phenomena seems impossible on a priori grounds, but because during more than twenty-five years of clinical observation directed especially toward these questions, I have never been cognizant of a single fact which would warrant such a conclusion. But I have been observing for many years, for long before I was in possession of any of the facts which I have been presenting in this paper, that the enamel on the rapidly decaying teeth of young persons often seems much harder and more resistant to the action of cutting instruments than the mature enamel on the slowly decaying teeth of adults. Several years ago I pointed out that there is a certain type of yellow tooth frequently seen in the mouths of German patients), which, although generally decaying very slowly, is a comparatively soft tooth.

My general conclusions are that all softening of enamel is due to the action of acids, and chiefly or wholly to the acids excreted by bacteria in situ; and that while soft enamel and rapid decay, or hard enamel and slow decay, may be observed coincidently, the apposite conditions may also be seen. In other words, although there are marked variations in the resistance of different enamels to cutting-instruments, these variations bear no necessary relation to decay or immunity from decay. But it is quite possible that observation demonstrates some relation between certain types of teeth and certain general systemic conditions which are favorable to the development and activity of the micro-organisms causing decay.

There are some very practical questions growing out of these conclusions, the most important of which has reference to the extent to which softening and decay of enamel may be prevented by the use of germicides. My own opinion, based on an experience extending over five years, is that we can place in the hands of our patients the means for preventing at least two-thirds of the dental decay now prevalent. This, of course, presupposes that the patients will do their part of the work well and faithfully. We have here, you will all admit, matter for serious consideration. In addition to the prophylactic measures suggested, the facts demonstrated call for more free cutting and thorough polishing of the enamel margins of cavities, and especially for the use of antiseptic varnishes and cements for the lining of all cavities.—*Dental Cosmos.*

